# Obituary

**Published:** 1855-09

**Authors:** 


*Obituary* —Dr. James C Bliss died in this city on the 30th of July, after having endured great suffering from Neuralgia, supervening upon a complication of maladies, which resisted all medication. Dr. B. was eminent as a practitioner, estimable as a man, distinguished as a Christian philanthropist, and in all relations of life he sustained an exalted character. No man in the profession, of New York, will be more missed by his brethren, or more deplored by his patients than he. He was 65 years of age, but retained his habits of activity, industry, and punctuality, which were proverbial, until smote down by his last illness. Peace to his memory.—*American Med. Gazette.*

